# Readability Metrics in Patient Education: Where Do We Innovate?

**DOI:** 10.3390/clinpract14060183

**Published:** 2024-11-04

**Authors:** Som Singh, Aleena Jamal, Fawad Qureshi

**Affiliations:** 1 Department of Internal Medicine, University of Missouri Kansas City School of Medicine, Kansas City, MO 64108, USA; 2 Sidney Kimmel Medical College, Thomas Jefferson University, Philadelphia, PA 19107, USA; 3 Department of Nephrology and Hypertension, Mayo Clinic Alix School of Medicine, Rochester, MN 55905, USA

**Keywords:** readability, patient education, opinion, improvement, research direction

## Abstract

The increasing use of digital applications in healthcare has led to a greater need for patient education materials. These materials, often in the form of pamphlets, booklets, and handouts, are designed to supplement physician–patient communication and aim to improve patient outcomes. However, the effectiveness of these materials can be hindered by variations in patient health literacy. Readability, a measure of text comprehension, is a key factor influencing how well patients understand these educational materials. While there has been growing interest in readability assessment in medicine, many studies have demonstrated that digital texts do not frequently meet the recommended sixth-to-eighth grade reading level. The purpose of this opinion article is to review readability from the perspective of studies in pediatric medicine, internal medicine, preventative medicine, and surgery. This article aims to communicate that while readability is important, it tends to not fully capture the complexity of health literacy or effective patient communication. Moreover, a promising avenue to improve readability may be in generative artificial intelligence, as there are currently limited tools with similar effectiveness.

## 1. Introduction

A tremendous rise in the use of digital applications in healthcare has marked this past decade. For clinicians, this includes growing innovation in the electronic medical record, which nuances clinical documentation and information dissemination [1,2]. Likewise, for patients, there has been increasing innovation in using digital access to medical information through provider–patient portals and the convenient dissemination of educational materials on patient ailments in both digital and non-digital modalities [3]. The latter educational materials are a critical component within the infrastructure of inpatient and outpatient patient education programs. Specifically, patients receive an array of pamphlets, booklets, and paper handouts that aim to educate them regarding their diseases and critical management points [4]. These aim to serve as supplementary sources of information for patients in addition to the patient’s physician, and they serve a significant role during situations where physician–patient communication is inadequate or there are important time constraints for physicians and healthcare professionals to educate a patient in the acute setting [4]. Educational materials are reported to contribute to positive patient outcomes and patient perception regarding their care [5]. However, these educational materials are static due to the varying levels of health literacy among the patients to whom they are provided. Patients with below-average health literacy have been shown to have worse hospitalization outcomes and spend more on prescriptions and healthcare utilization than those with higher health literacy, indicating an inverse relationship between health literacy and healthcare utilization [6,7].

### Readability

**Figure 1:**
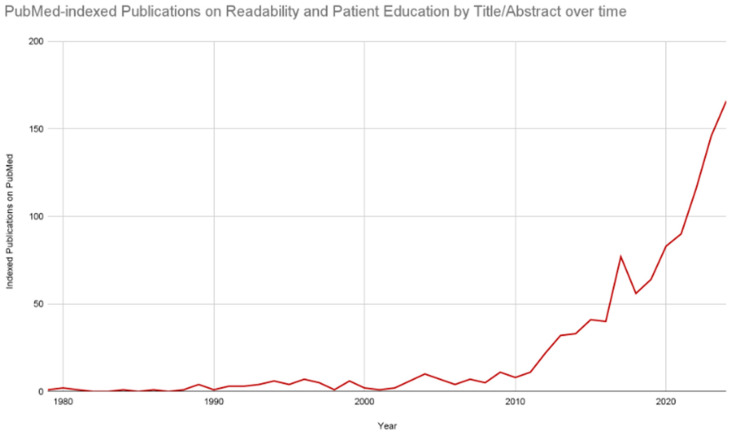
Publications on readability and patient education indexed in PubMed have increased over time. As of 8 August 2024. The employed query was (“readability”[Title/Abstract] AND “patient education”[Title/Abstract] AND “surgery”[Title/Abstract]).

Clinicians and researchers have explored the behaviors of patient health literacy, and within this concept is the comprehension quality of texts provided in the education materials to patients. While there are numerous contributors to the quality of text comprehension, readability is a prominent concept that the literature has often assumed to be associated with comprehension within this area [8,9]. Historically, readability was defined by George Klare as “the ease of understanding or comprehension due to the style of writing” [10]. When it comes to quantifying readability, it was the mid-19th century when the United States developed texts to be associated with grade-reading levels for students, and it has become a relatively common metric used by educators to assess student reading levels in the United States education system [9]. Eventually, the readability scoring will also become more frequent among adult civilians. These readability metrics were used in medicine, and the body of literature experienced tremendous growth in the 21st century, as demonstrated in Figure 1 [11,12,13].

The current readability metrics used to score texts are many, and DuBray reports that there may be over 200 readability formulas published [9]. In the current body of literature, many have often been used in medical education. The simple measure of gobbledygook (SMOG) score measure is a prominent readability formula with a reported high utility in consumer healthcare literature compared to other measures [14,15,16]. Wang et al. reported greater consistency between scores using the SMOG readability formula compared to other metrics [14]. However, these findings may be variable, as literature that has comparable readability metrics with a smaller area of medicine, such as a disease topic, has demonstrated no significant difference between readability metrics when compared to the SMOG [17,18]. It is, therefore, imperative to review the most prominent readability metrics studied in the literature. The Flesch Reading Ease Formula has also been prominently used in the clinical literature. It uses the number of sentences, syllables, and words within a scale of 1 to 100, where the higher the score, the greater the ease of reading the text sample is [19,20]. The Flesch Reading Ease score is one of the few unique metrics with this scale, as other metrics have been scored in a way that numerically correlates with a grade reading level. For example, the Flesch–Kincaid score factors in similar variables but can scale the score to approximate the grade reading level where an approximate score of 11.2 would suggest that the text is written at the level of an 11th-grade student. The Gunning Fog readability metric also uses a similar scaling but also considers words with three syllables or more as a variable. This grouping of words is considered “difficult” or “complex,” according to the total number of words with ≥3 syllables [9,21,22]. The New Dale–Chall readability metric employs a preselected list of words to categorize as another variable for words considered familiar and, therefore, calculated differently from words not included on this list [23].

**Figure 2:**
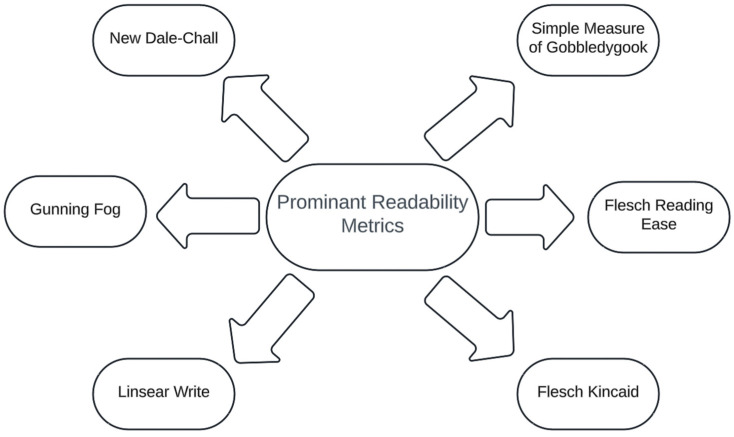
Readability metrics are frequently used in clinical literature. Of note, numerous readability metrics are designed that go beyond the scope of this figure.

As demonstrated in Figure 1, the body of literature on readability and patient education has accelerated over time across numerous aspects of patient communication. These metrics have demonstrated that research consent forms often need to meet the recommended sixth-to-eighth-grade reading level of texts [24]. These findings have been consistent across educational materials of interest, including consent forms, discharge paperwork, and online educational readings. Frequently, the published literature will suggest that patients’ education material must improve to make it easier for individuals to read, as the risk of literature with poor readability may indicate that patients will be more likely to comprehend information incorrectly, leading to misinformation [25,26,27]. The goal of this assertion is to explore the key components of literature that focuses on the readability of patient education materials (Figure 2).

## 2. Readability and Patient Education in Adult Internal Medicine

Online educational material on common inpatient admission diagnoses was also shown to have increased grade reading levels. Specifically, Hansberry et al. showed that resources had a mean readability over the 10th grade for conditions including pancreatitis, pulmonary embolism, and diverticulitis [28]. For individuals in geriatric care, the study by Weiss et al. primarily found that while the Health-in-Aging website aimed for simple language, its content was written at about a 10th-grade level [29]. Regarding patients in intensive and critical care settings, Hanci et al. reported that the mean Flesch reading ease score of online patient education materials from intensivist societies was over 50, indicating the text was “difficult to read” [15]. Still, no study has shown significant improvement to meet recommended grade reading levels. As a subset within internal medicine, educating patients with oncologic disease is imperative, as patients who undergo immunotherapy and radiation therapy require a great degree of longitudinal care and monitoring. The ability to maintain this longitudinal monitoring requires clinicians and oncology team members to educate patients on their condition appropriately. Moreover, Papadakos et al. report that oncology centers invest over USD 60,000 annually in developing educational pamphlets for patients alone [30]. Recent literature has demonstrated the use of large language models to produce educational texts on oncologic diseases [22,31,32]. These models have also been shown not to meet recommended reading levels. Similarly to literature in surgery, these generative artificial intelligence models have been able to improve reading levels. However, the improvement of these models was still unable to meet recommended grade reading levels, as they decreased text reading ease to the ninth grade. Likewise, online education materials for adult endocrine diseases were reported to have a range of Flesch Reading Ease scores ranging between “fairly difficult to read” and “very difficult to read” [21]. This poor readability was similarly demonstrated in numerous studies regarding rheumatologic condition as well, exceeding the eighth-grade reading level [11,16].

## 3. Readability and Patient Education in Pediatric Medicine

The relationship between pediatric medical teams and their patients is unique compared to other populations. Patient education will require communication with both the parents at an appropriate level and the patients, given their age [33]. Similarly, readability metrics on patient education materials are often found to be too advanced for patients and have even been shown to be a barrier to the enrollment of patients in research studies. However, a study in 2005 showed that some education brochures by the American Academy of Pediatrics were acceptably low enough in grade reading level [34]. There is a relative paucity of more recent, longitudinal results. These are essential given the tremendous growth of online literature since the publication of this study. Okuhara et al. published a systematic review of the readability of online and offline vaccine informational materials [35]. Some of the extracted studies on childhood vaccinations reported both a poor reading status by the parents of the patients and poor reading materials.

## 4. Readability and Patient Education in Preventative Medicine

There is a growing interest in preventative healthcare among individuals as chronic diseases remain a primary catalyst in healthcare utilization costs [36]. Despite this, there is a stasis in readability for many preventative measures. For example, coronary artery calcium scans were demonstrated in a 2020 study to have an overall mean readability score of 10.9 for public online educational materials [37]. However, a 2023 study demonstrated a mean Flesh–Kincaid score of 11.3, which may indicate no change or worse readability over this period [38]. A study by Skrzypczak and Mamak observed that the readability of websites with information regarding colonoscopy was classified as “very hard to comprehend” [39]. The mean Flesch–Kincaid score for lung cancer screening is over the recommended reading levels, and approximately over the 10th-grade reading level [40]. This above-recommended level was also found in breast-cancer-screening educational materials [41]. Gu et al. reported a systematic review and meta-analysis of online patient education materials on breast cancer and found inconsistently reported readability metrics in this body of literature [42]. However, most of this analysis did not meet recommended grade reading levels. AlKhalili et al. showed that 42 internet-based education materials on mammography and breast-cancer screening had a mean Gunning Fog score of over 14, indicating a grade reading level nearly twice as high as the recommended levels [43]. Parry et al. reported a mean readability of 9.5 for the readability of online articles regarding pap smears [44]. For common medications, Ngo et al. found that online educational materials on statins have a mean readability score over 10 [45].

## 5. Readability and Patient Education in Surgery

Within surgery, informed consent plays one of the most vital roles in patient care. Physicians and surgical-team members must assess a patient’s health literacy regarding their disease and management options [46]. Lin et al. reported a population of procedure consent forms from 104 hospitals in the United States. This study discovered that the mean grade reading level of these procedure consent forms was 11th grade and did not meet the recommended grade reading levels [47]. Peer-reviewed articles have observed readability metrics using digital and internet-based articles regarding numerous surgical aspects (i.e., neurological surgery, gastrointestinal surgery, plastic surgery, sinus surgery, and trauma surgery) [48,49,50,51,52,53,54]. These studies have reported poor readability levels of the educational materials employed. Furthermore, the readability of content from national organizations and high-impact articles showed no difference in meeting recommended grade reading levels [11,50]. Moreover, an article by Zhang et al. reports that there has been limited improvement in the readability of online patient educational materials regarding hand surgery in 2008 and 2015 [49]. The more recent body of literature regarding surgical patient education and readability since 2020 has included observing the behavior of readability in the setting of generative artificial intelligence [32]. An article by Ali et al. demonstrated the ability to use a generative pre-trained transformer (GPT) to improve the readability of surgical consent forms [55]. Moreover, this study showed that post-GPT, readability improved from the college level to the eighth-grade reading level.

## 6. Discussion

Readability metrics may not give a complete picture of comprehension. All sections reported in this review demonstrated limited mean readability levels that do not meet the recommended reading level between sixth and eighth grades. Rather, readability may be a much more nuanced, personalized score that cannot be entirely encompassed by a grade reading level. Moreover, scores such as the Flesch Reading Ease and Flesch–Kincaid do not account for the subjective matter of which words are familiar to readers. This reading familiarity may not be weighted similarly to unfamiliar words [56]. Although the Dale–Chall and New Dale–Chall metrics aim to provide a word list to account for familiarity, these metrics may also not account for words that may be more familiar for patients within topic subsets [23,57]. This creates an opportunity for clinicians and researchers in readability to focus on developing a metric that also accounts for readable familiarity specific to a subject topic. For example, patients undergoing dialysis may have a greater familiarity with words regarding their treatment compared to patients with no reported history of renal diseases.

The concern for readability criteria among many cross-sectional studies has been reported in prior literature. However, there seems to be a paucity in the lack of transparent ways to improve the readability of text given the cross-sectional design of many of these studies. Hackos and Stevens describe a number of ways to improve texts that may be able to be employed, including avoiding jargon, using the present tense, and using culture- and gender-neutral languages [58]. Moreover, researchers ought to investigate the ability to design innovative tools that can review texts and suggest these readability simplification factors. One of these tools may be in generative artificial intelligence given the demonstrated reduction in readability scores.

Generative artificial intelligence models demonstrated an ability to decrease readability scores when applied to these models [22,59,60]. This is a promising avenue to improve readability, given that the longitudinal improvements across numerous online medical contents have not demonstrated statistically significant improvement to meet recommended grade reading levels over time. Although grade reading level recommendations were not met by many studies that implemented generative artificial intelligence, it is vital to understand that the literature on using these models continues to grow in the literature. The models will eventually utilize their deep learning attributes to adapt readability. However, it remains imperative that these models are monitored longitudinally over time, as deep learning may also result in artificial intelligence hallucination, impairing our understanding of proper health literacy and readability.

This opinion article is limited in its scope where it focused on four major avenues of medical care: surgery, adult medicine, pediatric medicine, and preventative medicine. Likewise, not all subspecialties or medical topics were discussed. However, the depth of readability studies all demonstrated the similar finding that texts do not meet recommended grade reading levels. Instead, this study provides an updated status on the current state of the literature, indicating that promising avenues to improve readability may be found in the use of generative artificial intelligence models. These models may be used across numerous aspects of medicine. The decision to design this opinion article around these four areas in medicine is because they cover common areas of outpatient medical care that patients obtain. Likewise, this review also notes the need for more investigation on readability in pediatric medicine, as there is a lack of literature in that area compared to internal medicine and surgical fields.

## 7. Conclusions

Many studies report similar findings showing that their samples of readable texts do not meet the recommended sixth-to-eighth grade reading level. A possible area to explore may be understanding that readability alone may not be a metric appropriate to determine health literacy or patient communication. Instead, it demonstrates that texts urgently need to improve. One promising avenue for improvement may be in training generative artificial intelligence, as there is a lack of tools in the literature that have shown a similar degree of readability improvement. Another possible avenue may be to improve readability scoring using metrics that employ updated or more personalized strategies, like the New Dale–Chall formula, which has an updated list of familiar words that are weighted as a different variable. This may help improve our understanding of readability at a more personalized level.

## Data Availability

Not applicable.
